# Overexpression of the proneural transcription factor ASCL1 in chronic lymphocytic leukemia with a t(12;14)(q23.2;q32.3)

**DOI:** 10.1186/s13039-018-0355-7

**Published:** 2018-01-11

**Authors:** Theodora Malli, Melanie Rammer, Sabrina Haslinger, Jonathan Burghofer, Sonja Burgstaller, Hans-Christian Boesmueller, Renate Marschon, Wolfgang Kranewitter, Martin Erdel, Sabine Deutschbauer, Gerald Webersinke

**Affiliations:** 1 Laboratory for Molecular Biology and Tumor Cytogenetics, Department of Internal Medicine I, Ordensklinikum Linz GmbH Barmherzige Schwestern, Seilerstaette 4, 4010 Linz, Austria; 2 Department for Internal Medicine IV, Klinikum Wels-Grieskirchen, Grieskirchner Str. 42, 4600 Wels, Austria; 3 Department of Pathology, Ordensklinikum Linz GmbH Barmherzige Schwestern, Seilerstaette 4, 4010 Linz, Austria; 4 Present Address: Institute for Pathology and Neuropathology, University Hospital Tuebingen, Liebermeisterstraße 8, 72076 Tuebingen, Germany

**Keywords:** *ASCL1* overexpression, *INSM1*, *IGH* translocation, Chronic lymphocytic leukemia, Expression microarray

## Abstract

**Background:**

Translocations of the *IGH* locus on 14q32.3 are present in about 8% of patients with chronic lymphocytic leukemia (CLL) and contribute to leukemogenesis by deregulating the expression of the *IGH*-partner genes. Identification of these genes and investigation of the downstream effects of their deregulation can reveal disease-causing mechanisms.

**Case presentation:**

We report on the molecular characterization of a novel t(12;14)(q23.2;q32.3) in CLL. As a consequence of the rearrangement *ASCL1* was brought into proximity of the IGHJ-Cμ enhancer and was highly overexpressed in the aberrant B-cells of the patient, as shown by qPCR and immunohistochemistry. *ASCL1* encodes for a transcription factor acting as a master regulator of neurogenesis, is overexpressed in neuroendocrine tumors and a promising therapeutic target in small cell lung cancer (SCLC). Its overexpression has also been recently reported in acute adult T-cell leukemia/lymphoma.

To examine possible downstream effects of the *ASCL1* upregulation in CLL, we compared the gene expression of sorted CD5^+^ cells of the translocation patient to that of CD19^+^ B-cells from seven healthy donors and detected 176 significantly deregulated genes (Fold Change ≥2, FDR *p* ≤ 0.01). Deregulation of 55 genes in our gene set was concordant with at least two studies comparing gene expression of normal and CLL B-lymphocytes. *INSM1*, a well-established ASCL1 target in the nervous system and SCLC, was the gene with the strongest upregulation (Fold Change = 209.4, FDR *p* = 1.37E-4).

*INSM1* encodes for a transcriptional repressor with extranuclear functions, implicated in neuroendocrine differentiation and overexpressed in the majority of neuroendocrine tumors. It was previously shown to be induced in CLL cells but not in normal B-cells upon treatment with IL-4 and to be overexpressed in CLL cells with unmutated versus mutated *IGHV* genes. Its role in CLL is still unexplored.

**Conclusion:**

We identified *ASCL1* as a novel *IGH*-partner gene in CLL. The neural transcription factor was strongly overexpressed in the patient’s CLL cells. Microarray gene expression analysis revealed the strong upregulation of *INSM1*, a prominent *ASCL1* target, which was previously shown to be induced in CLL cells upon IL-4 treatment. We propose further investigation of the expression and potential role of *INSM1* in CLL.

**Electronic supplementary material:**

The online version of this article (10.1186/s13039-018-0355-7) contains supplementary material, which is available to authorized users.

## Background

Chronic lymphocytic leukemia (CLL) is characterized by the accumulation of small clonal mature B-lymphocytes in blood, bone marrow (BM) and lymphatic tissues [1]. CLL cells present with a distinctive immunophenotype defined by the co-expression of CD5, CD19 and CD23. The levels of surface immunoglobulin, CD79b and CD20 are low compared to normal B-lymphocytes [2]. The clinical course of CLL is heterogeneous, ranging from long-term survival without the need of treatment to rapid progression despite early and aggressive therapy.

Recurrent cytogenetic lesions are found in more than 80% of the CLL patients and have a prognostic value. Deletions are mostly found at 13q, followed by 11q, 17p and 6q, while trisomy 12 is the most common numerical aberration [3, 4]. Although translocations occur in about 32–34% of the CLL cases, recurrent chromosomal translocations are rare events, found in about 5% of the patients [5, 6]. Most translocation breakpoints cluster on 13q14 followed by the *IGH* locus on 14q32.3 [4, 5]. A recent review of 18 studies estimated the overall frequency of *IGH* rearrangements in CLL to be about 8%, with reported frequencies varying between 2 and 26% [7].

*IGH* rearrangements can occur during *IGH* locus remodeling as a result of VDJ recombination, somatic hypermutation or class switch recombination. All these procedures take place in the course of B-cell development and involve the generation and re-ligation of double strand breaks [8]. *IGH* locus breakpoints cluster in the joining (*IGHJ*) and switch regions (*IGHS*) [9], although breakpoints in the variable (*IGHV*) and diversity (*IGHD*) regions have also been described [10]. In most instances, the biological consequence of the rearrangement is the deregulation of the partner gene, due to its juxtaposition to one of the *IGH* enhancers, reviewed by Willis and Dyer [11]. Except of the t(14;18)(q32;q21), immunoglobulin gene translocations are associated with a poor prognosis in CLL [7].

Here we report on the molecular characterization of a novel t(12;14)(q23.2;q32.3) in a patient with CLL. A search in the Mitelman Database of Chromosome Aberrations and gene fusions in cancer [12] for translocations involving the 12q23 region in CLL patients revealed three further cases reported in the literature [6, 13, 14]. Molecular characterization was performed in only one of these cases and revealed a fusion of the *CHST11* gene on 12q23.3 to the *IGH* locus [13].

## Case presentation

Our patient was a 58-year old female, diagnosed with CLL in 2002. Abnormal lymphocytes showed expression of CD5, CD19, CD20, CD22, CD23 and immunoglobulin kappa light chain by flow cytometry. Ubiquitous enlarged lymph nodes were detected. The patient was asymptomatic. First line treatment was required 2003 due to increasing leukocytosis and lymphocytosis accompanied by advancing anemia and thrombocytopenia. The patient was treated with chlorambucil and prednisone (Knospe protocol) according to local standards and therapeutic possibilities at that time. After achieving a partial remission persisting approximately one year, the patient was retreated with continuous chlorambucil for one month but showed no response. Four cycles of oral fludarabine were administered achieving a partial remission for four years. The following two relapses of the disease were treated again with fludarabine, of which the latter course was mainly due to patient’s preference. After documenting resistance to fludarabine the patient agreed to administration of five cycles rituximab in combination with bendamustine. A partial remission could be achieved. Rituximab and bendamustine were used for treating the following relapse 1.5 years later, achieving a partial remission for another eight months. Afterwards the patient received ibrutinib within a clinical trial, but showed progression of disease after only four months of treatment. Massive progression of lymphadenopathy was apparent at that time. Therefore, a lymph node biopsy was done showing a diffuse infiltration with small lymphocytic cells partially resembling centroblasts or immunoblasts, though transformation to an aggressive lymphoma could not be demonstrated. According to the clinical behavior of the disease, rituximab plus CHOP were administered but progression occurred after three cycles of treatment. Alemtuzumab was then administered achieving stabilization of the disease for another year. Ultimately, the patient was treated with lenalidomide but showed no significant response and died 2014 due to pneumonia. Informed consent for studies performed and for publication of the results was obtained from the patient. All methods used are described in detail in Additional file 1.

**Fig. 1:**
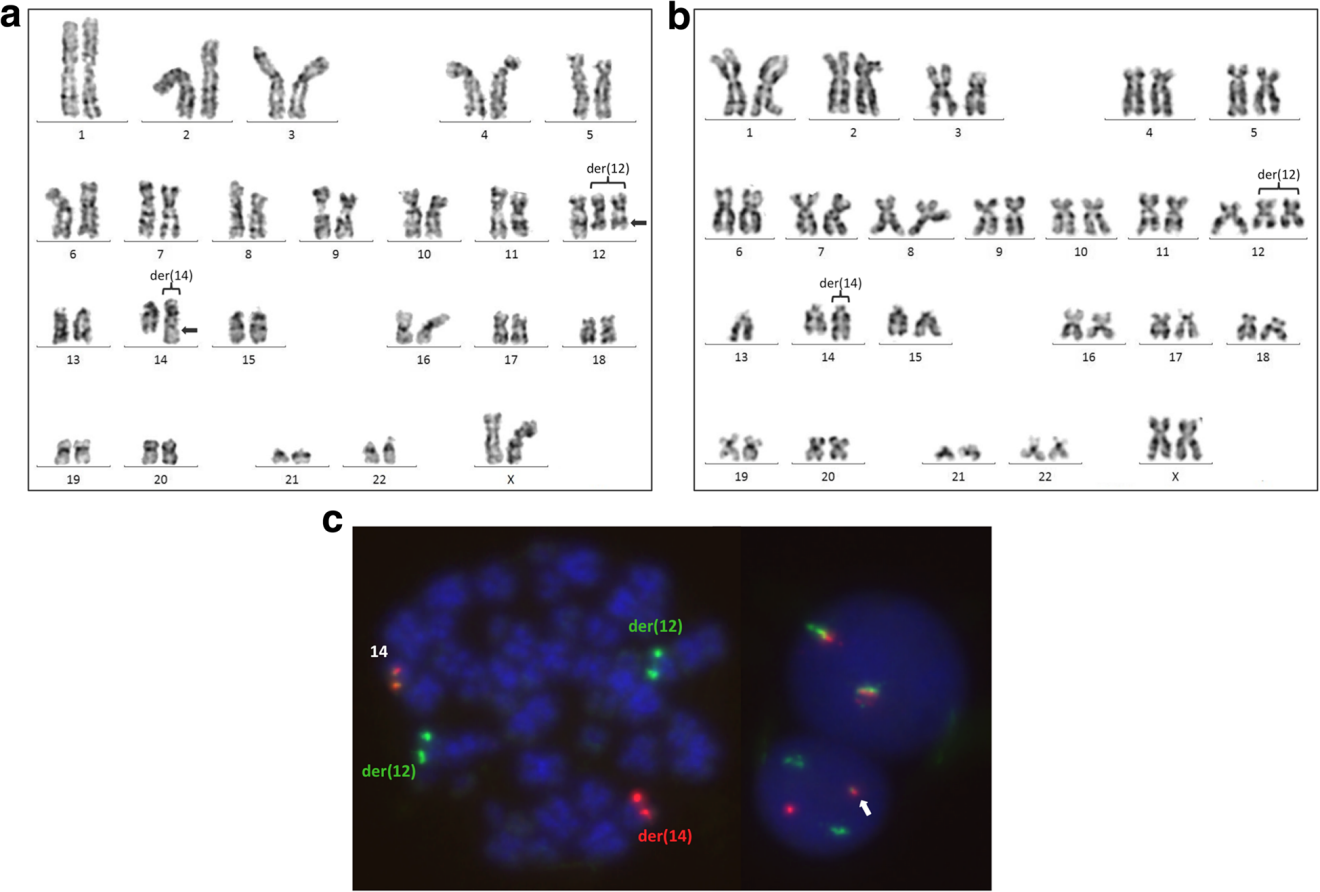
**a** Karyotype of the patient displaying the t(12;14)(q23.2;q32.3). Arrows mark the translocation breakpoint regions on the derivative chromosomes. Note that der(12) is duplicated, leading to a partial trisomy 12. **b** Karyotype evolution (about three years later). Additional aberrations include a del(3)(p21), monosomy 13 and add(17)(p11). For detailed information see also Table 1. **c** FISH with the Cytocell IGH Breakapart probe on metaphase and interphase nuclei. The normal chromosome 14 generates a red-green fusion fluorescence signal. Der(14) yields only a red fluorescence signal with the distal green-labeled probe being translocated on der(12). A second green fluorescence signal is present due to the der(12) duplication. On the upper right side, a normal interphase with two red-green fusion signals is shown, next to an interphase bearing the translocation (lower right). A white arrow marks the fusion signal from the normal chromosome 14

Patient material was first sent to our laboratory eight years after the initial diagnosis of CLL. In the next four years, karyotyping and FISH studies were performed seven times in intervals of six to twelve months. The detailed cytogenetic findings in the seven samples of the patient, analyzed between 2010 and 2014, are summarized in Table 1. Consistent findings in all patient probes included the t(12;14)(q23.2;q32.3), a partial trisomy 12 due to duplication of der(12) chromosome (Fig. 1a) and a submicroscopic deletion of the 13q14 region. Signal splitting of the Cytocell IGH Breakapart probe confirmed the involvement of the *IGH* locus on chromosome 14 in the translocation (Fig. 1b). The duplication of der(12) indicates that the t(12;14)(q23.2;q32.3) preceded trisomy 12. Since trisomy 12 is considered to be an early driver clonal event in CLL [15], we propose that the translocation occurred early in CLL evolution. Nevertheless, it is not possible to experimentally confirm that, since no sample was available at the time of diagnosis.

**Table 1:** Summary of the cytogenetic findings in the seven samples of the patient

Date	Cytogenetic Findings	Bone Marrow (BM) Infiltration, Therapy
27.05.2010	47,XX,t(12;14)(q23.2;q32.3),+der(12)t(12;14)[9]/46,XX[11]. nuc ish(ATM,TP53)×2[204],(D12Z1x3,D13S319x1,LAMP1×2)[158/230],(5’IGHx3,3’IGH×2)(5’IGH sep 3’IGHx1)[167/216]	80% BM infiltration, fludarabine resistance, thrombopenia, therapy switch to rituximab and bendamustine
21.03.2011	47,XX,t(12;14)(q23.2;q32.3),+der(12)t(12;14)[7].ish der(12)t(12;14)(q23.2;q32.3)x2(5’IGH+). nuc ish(D12Z1x3,D13S319x1,LAMP1x2)[91/212], (5’IGHx3,3’IGHx2)(5’IGH sep 3’IGHx1)[91/223]	Thrombocytopenia, 40% BM infiltration
24.10.2011	47,XX,t(12;14)(q23.2;q32.3),+der(12)t(12;14)[17]/46,XX[5]	Rituximab and bendamustine
14.03.2012	47,XX,t(12;14)(q23.2;q32.3),+der(12)t(12;14)[19]/46,XX[1]	95% BM infiltration
11.03.2013	45~47,XX,del(3)(p21),t(12;14)(q23.2;q32.3),+der(12)t(12;14),-13,add(17)(p11.2)[cp8]/46,XX[3]. nuc ish(ATMx2,TP53x1)[121/169],(D12Z3x3,D13S319x0,LAMP1x1)[146/217]/ (D12Z3x3,D13S319x1,LAMP1x1)[17/217]	85% BM infiltration, progression despite therapy with Ibrutinib, lymphadenopathy
03.09.2013	47,XX,t(12;14)(q23.2;q32.3),+der(12)t(12;14)[5]/46,idem,del(3)(p21),-13,add(17)(p11.2)[3]/46,XX[3]	80% BM infiltration, Alemtuzumab
09.01.2014	47,XX,t(12;14)(q23.2;q32.3),+der(12)t(12;14)[7]/46,idem,del(3)(p21),-13,add(17)(p11.2)[2]/46,XX[6]	96% BM infiltration, sample for expression microarray received

Clinical findings and therapy at the same time points are also stated

**Fig. 2:**
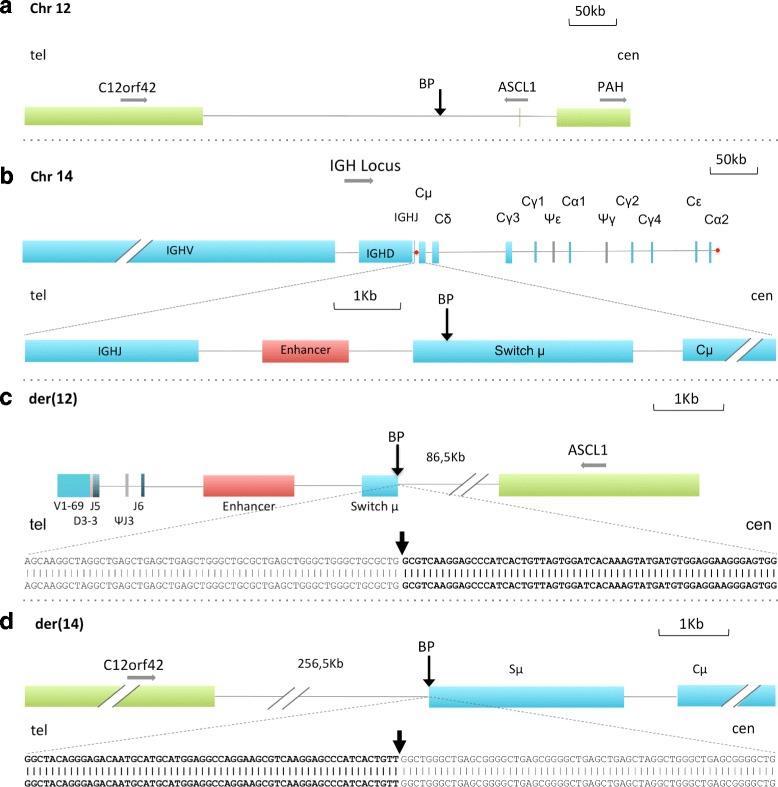
Translocation breakpoints and derivative chromosome composition. Horizontal gray arrows indicate the transcriptional direction of the depicted genes. Vertical black arrows indicate breakpoints (BP). **a** Breakpoint region on chromosome 12. The breakpoint took place 86.5 kb distal of the *ASCL1* gene. **b** The *IGH* locus on chromosome 14. The breakpoint took place within the pentameric repeat region of Switch μ. Dots indicate the *IGH* enhancer elements. **c** Composition of der(12) and sequence around the breakpoint. The enhancer element is part of the translocated *IGH* sequence and is juxtaposed to *ASCL1*. **d** der(14) and breakpoint sequence. The *C12orf42* gene is translocated to chromosome 14

Sequencing of LDI-PCR-generated *IGHJ* bands varying from the expected germline bands revealed a productive VDJ recombination with an unmutated V1–69 gene (100% sequence homology) fused to D3–3 and J5 sequences and a D-J recombination between D2–21 and J5 on the other allele. Sequencing of the aberrant *IGHS* bands revealed sequences from chromosome 12 integrated into the Switch μ (Sμ) region. A second round of sequencing with a reverse primer from chromosome 14 (IGH der12 Rv) was necessary to read over the breakpoint on der(12), which was located 86.5 kbp downstream of the achaete-scute family bHLH transcription factor 1 (*ASCL1*) gene*.* Primer sequences are listed in (Additional file 2: Table S1). The *IGHJ*-*Cμ* enhancer was translocated in the proximity of *ASCL1*, while the more distal gene *C12orf42* was translocated to der(14). The breakpoint on der(14) was localized within the pentameric repeat region of Sμ. There were no deletions or insertions of sequences at the breakpoints of both chromosomes (Fig. 2).

**Fig. 3:**
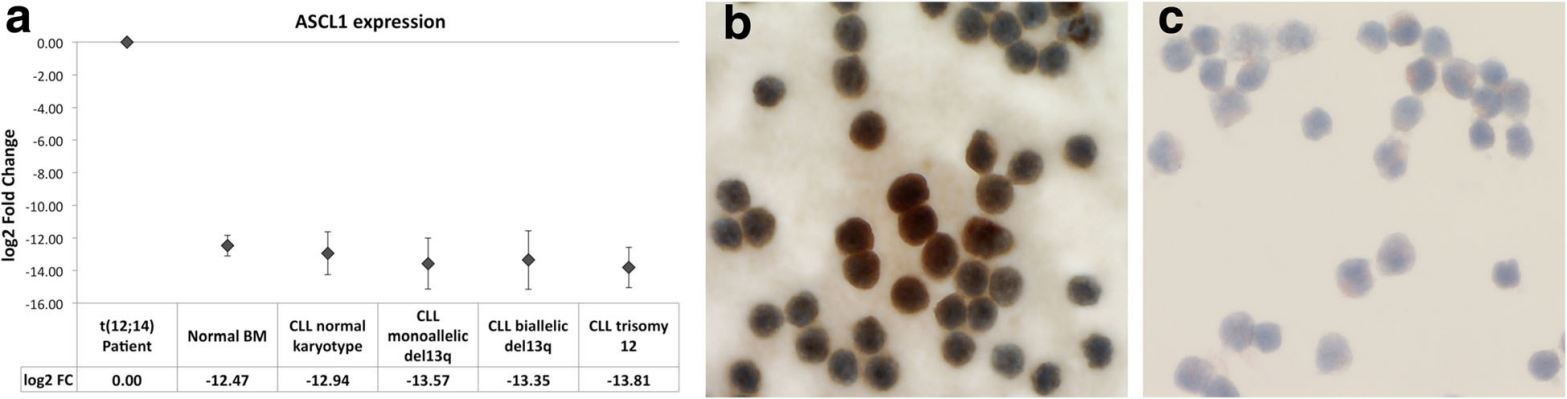
Validation of the *ASCL1* overexpression. **a** Comparison of the BM expression of *ASCL1* between the CLL patient with the t(12;14) translocation and healthy controls as well as CLL with normal karyotype, mono- and biallelic del(13) and trisomy 12 respectively. Results are displayed as log2 fold change. *HB2M* was used as housekeeping control. Comparisons of the *ASCL1* expression in the patient versus all other groups were highly significant (ANOVA *p*-value = 5.12E-10). Comparisons between normal BM and all other groups were not significant. **b** Immunohistochemistry for ASCL1 on a peripheral blood cytospin of the patient bearing the t(12;14). Note the strong nuclear reaction in the center. In contrast to that a sample from a B-CLL control (**c**) showed no antibody reaction. Nuclei are counterstained with hematoxylin

The expression of *ASCL1* in the BM of the patient bearing the translocation (90% infiltration) was compared to that in normal and CLL BM samples (mean infiltration >70%). CLL samples were subdivided in four groups according to their cytogenetic findings (Table 2). *ASCL1* was highly overexpressed in the sample of the patient bearing the translocation as opposed to all other groups with average fold change (FC) values greater than 5600 in all samples (ANOVA *p*-value = 5.12E-10) (Fig. 3a). Immunohistochemistry with a monoclonal anti-ASCL1 antibody on peripheral blood cytospins of the patient and two CLL control samples confirmed the ASCL1 overexpression at the protein level (Fig. 3b and c).

**Table 2:** CLL patient samples used for *ASCL1* quantification by qPCR

	Normal cytogenetics	Monoallelic Del(13q)	Biallelic Del(13q)	Trisomy 12
Sample number	7	8	6	9
*IGHV* hypermutated/unmutated	3/4	4/4	3/3	6/3
Mean aberrant cells in BM	81%	88%	88%	72%

Distribution according to cytogenetic findings, *IGHV* mutation status and mean bone marrow (BM) infiltration

*ASCL1,* also known as *hASH1* or *mASH1,* is the human homolog of the Drosophila achaete-scute complex. It encodes for a basic pioneer helix-loop-helix transcription factor (TF), which is a master regulator of vertebrate neurogenesis [16]. In order to further explore the possible downstream effects of the *ASCL1* upregulation in the aberrant B-cells of the patient, we compared the gene expression of these cells to that of sorted B-cells from seven healthy donors, using the GeneChip® PrimeView™ Human Gene Expression Array (Affymetrix, Santa Clara, CA). We found 176 significantly deregulated genes (FC ≥ 2, FDR *p* ≤ 0.01) (Additional file 3: Figure S1) and (Additional file 4: Table S2). Deregulation of 55 genes in our gene set was concordant with at least two CLL expression studies comparing CLL cells to peripheral CD19^+^ B-lymphocytes of healthy individuals (see also Additional file 4) [17–20].

We then focused on the genes with the strongest deregulation in our gene set (FC ≥ 10, FDR *p* ≤ 0.001) (Table 3). Seven of the top 18 deregulated genes (*ABCA9, KCNJ11, FHDC1, KSR2, EBF1* and *RGS2*) were part of the above-mentioned CLL gene expression signature. The deregulation of three further genes from this list (*RGS1, APP*, *GABRB2* and *FGF2*) was concordant with CLL versus normal comparisons from the Oncomine Database [21–24]. Among the residual eight highly deregulated genes the overexpression of *ASCL1* and also *PAH,* localized 40 kbp proximal to the *ASCL1* locus, could be explained by their proximity to the *IGH* enhancer due to the translocation. *PAH* encodes for phenylalanine hydroxylase, an enzyme involved in phenylalanine catabolism. To our knowledge, no oncogenic properties have been assigned to the *PAH* gene so far. Binding of ASCL1 on promoter sequences of the *MRO*, *EDNRB* and *RNF150* genes has been demonstrated by ChIP in adult hippocampus-derived neural stem cells [25]. The overexpression of *GLDN* and *PAX9* has not been previously described in CLL and these genes are also not listed among the direct *ASCL1* targets. *INSM1*, the gene with the highest upregulation and the third most significant in our gene set, is a well- established direct ASCL1 transcriptional target in neural and neuroendocrine tissue as well as in SCLC [26–28].

**Table 3:** Highly deregulated genes in the CLL cells of the translocation patient versus normal B-cells (FC > 10, FDR *p* < 0.01)

Gene Symbol	Fold Change	FDR *p*-value	Deregulation in CLL	Reference
*INSM1*	209.42	0.000137		
*ASCL1*	202.32	0.000029		
*ABCA9*	118.18	0.000114	overexpressed	[17] [19] [20]
*PAH*	84.87	0.000166		
*MRO*	82.52	0.000408		
*GABRB2*	42.47	0.000495	overexpressed	[21]
*PAX9*	38.71	0.000800		
*RNF150*	27.45	0.000748		
*FHDC1*	25.51	0.000537	overexpressed	[17] [19] [20]
*KCNJ11*	20.52	0.000458	overexpressed	[19] [20]
*KSR2*	20.37	0.000748	overexpressed	[17] [20]
*FGF2*	18.37	0.000748	overexpressed	[22]
*GLDN*	18.02	0.000881		
*EDNRB*	12.07	0.000495		
*APP*	−21.43	0.000489	underexpressed	[21]
*RGS2*	−24.38	0.000902	underexpressed	[17] [19]
*EBF1*	−61.42	0.000976	underexpressed	[17] [19]
*RGS1*	−98.36	0.000970	underexpressed	[17] [23]

Information about gene deregulation in CLL and relevant references are shown

## Discussion and conclusions

We report on a CLL patient bearing a t(12;14)(q23.2;q32.3). So far, molecular characterization of one CLL case with a t(12;14)(q23;q32) has been reported in the literature [13]. The chromosome 12 breakpoint was located about 1.4 Mb distal to that found in our patient and disrupted the *CHST11* gene encoding for a Golgi-associated sulfotransferase. The translocation probably led to the expression of truncated versions of the CHST11 protein with altered cellular distribution [13].

In the present case, the translocation led to the overexpression of *ASCL1* and the more proximal *PAH* gene in the aberrant B–cells of the patient. *ASCL1* plays a role in the development of lung neuroendocrine cells [29], thyroid C cells [30] and adrenal chromaffin cells [31], is overexpressed in neuroendocrine tumors [32] and is a promising therapeutic target in SCLC [27, 33]. Several transcriptional targets of ASCL1 have been identified in normal neural development and in cancer cells with functions in NOTCH signaling, cell proliferation and differentiation [25, 27, 33–37]. It is remarkable that ASCL1 acts as a pioneer TF, having the ability to access nucleosomal DNA, promote its opening and accessibility to other TFs [36, 38, 39] and enable reprogramming non-neural cells to induced neurons [40, 41].

According to a meta-analysis of microarray data in the Oncomine database, *ASCL1* was one of the top 1% overexpressed genes in acute adult T-cell leukemia/lymphoma (FC: 3.76, *p* = 3.43E-5) [24, 42, 43], while reduced expression of *ASCL1* was reported in diffuse large B-cell, primary effusion and mantle cell lymphoma [24, 43]. The biological consequences of the above observations are currently unknown. According to the same database, a study comparing the expression profiles of normal and CLL peripheral mononuclear cells reported underexpression of *ASCL1* in CLL (FC = −3.07 *p* = 5.31E-4) [24, 44]. Nevertheless, this could not be confirmed by a study with a larger patient cohort, comparing the same cell types [21, 24]. According to our qPCR results, there were no significant *ASCL1* expression differences between normal BM and that of various CLL cytogenetic subsets (mean BM infiltration >70%) (Fig. 3).

Global gene expression analysis of the patient’s CLL cells versus B-cells from healthy donors revealed a CLL gene expression signature comprising of 55 genes, concordant with published results of at least two studies comparing the same cell types. *INSM1*, the gene with the highest fold change in the patient, is a prominent ASCL1 target [26, 27, 33, 35, 45]. It is likely that its strong deregulation in the B-cells of our patient is a result of the *ASCL1* overexpression. Nevertheless, since the targets of a transcription factor can vary depending on the cellular context, it is not possible to exactly predict which genes would actually be regulated by ASCL1 in a B-cell without performing functional studies.

*INSM1* encodes for a conserved zinc-finger transcriptional repressor [46], which controls neuroendocrine differentiation and is overexpressed in the majority of neuroendocrine tumors [26, 47]. Notably, INSM1 is also able to exert its function by directly influencing signaling pathways through protein-protein binding. For example, its association with cyclin D1 (CCND1) has been reported to cause cell cycle lengthening without triggering apoptosis [48].

Little is known about the potential role of *INSM1* in CLL. According to Liao et al. 2015 *INSM1* expression is higher in CLL cells with unmutated versus that with mutated *IGHV* genes [17]. Ruiz-Lafuente et al. reported induction of *INSM1* in CLL cells but not in normal B-cells upon treatment with IL-4 [17]. Since IL-4 stimulation is part of the stromal interactions that protect CLL cells from apoptosis, genes induced by IL-4 in CLL cells could contribute to their survival [17]. The *INSM1* overexpression in the peripheral B-cells of our patient, possibly taking place due to the *ASCL1* overexpression, could provide a further hint for a potential role of *INSM1* in CLL, thus we propose the further examination of its expression and possible role in CLL pathogenesis.

## Additional files

Additional file 1: Material and Methods. (DOCX 149 kb)

Additional file 2: Table S1. Primer Sequences. (DOCX 120 kb)

Additional file 3: Figure S1. Hierarchical Clustering. (DOCX 115 kb)

Additional file 4: Table S2. List of deregulated genes (Fold Change ≥2, FDR *p* ≤ 0.01). (DOCX 474 kb)

## Abbreviations

BM: Bone marrow
CLL: Chronic lymphocytic leukemia
FC: Fold change
SCLC: Small cell lung cancer
TF: Transcription factor
