# Supplementary material for: Overexpression of the proneural transcription factor ASCL1 in chronic lymphocytic leukemia with a t(12;14)(q23.2;q32.3)
Source: Mol Cytogenet. 2018 Jan 11;11:3. doi: 10.1186/s13039-018-0355-7 (PMC5765657; doi:10.1186/s13039-018-0355-7)
Supplement: Supplementary file 1 — Material and Methods. (DOCX 149 kb) [file 13039_2018_355_MOESM1_ESM.docx]

Additional File 1: Material and Methods

*Karyotyping and FISH experiments*

Conventional karyotyping was performed on IL-2/DSP30 (Sigma Aldrich, St. Louis, MO) stimulated and non-stimulated bone marrow (BM) cultures. FISH experiments for detection of the most frequently described chromosomal aberrations in B-CLL were performed on the same material with following commercial probes: Vysis LSI TP53 (17p13.1)/LSI ATM (11q22.3) and LSI D13S319/ LSI 13q34/ CEP 12 Multi-color Probe Set (Abbott Molecular, Des Plaines, IL), MYB (6q23)/D6Z1 dual color and IGH Breakapart (CytocellP^TM^P, Cambridge, UK).

*LDI-PCR and sequencing*

Long distance inverse PCR (LDI-PCR) followed by nested PCR was used to amplify the breakpoint region on der(12) as already described [1] with primers for the *IGHJ* [2] and *IGHS* regions [3]. The nested PCR products were subjected to agarose electrophoresis. Bands not corresponding to the expected germline length were purified with the peqGOLD gel extraction kit (Peqlab, Erlangen, Germany) amplified with the same primers and sequenced. A second round of sequencing with primer IgH der12 Rv was necessary to read over the breakpoint on der(12). Primers IgH der14 Fw and IgH der14 Rv were used to amplify and sequence the breakpoint on der(14). For primer sequences please refer to the supporting information (Supplementary Table S1).

*Quantitative reverse transcriptase PCR*

The *ASCL1* expression in the BM of the patient with the t(12;14)(q23.2;q32.3) was compared to that of 29 CLL patients and 10 normal BM samples. Details about the cytogenetic findings, the *IGHV* mutation status and the extent of BM infiltration of the CLL patients are summarized in Table 2.

Reverse transcription was performed with the M-MLV Reverse Transcriptase, RNase H Minus, Point Mutant 200 U/µl by random hexamer priming (Promega, Madison, WI) in duplicates. The TaqMan™ gene expression assay Hs00269932_m1 for *ASCL1* and the human beta-2-microglobulin (*B2M*) Endogenous Control 4310886E (Applied Biosystems, Foster City, CA) were used for relative quantification of the *ASCL1* expression in duplicate reactions. Evaluation of relative expression differences was accomplished using the comparative Cq method, Welch’s ANOVA and Tukey-HSD/Tukey-Kramer post-hoc analysis [4].

*Immunocytochemistry*

Immunocytochemistry was performed according to standard procedures on peripheral blood cytospins of the patient and two CLL controls using a 1:50 dilution of a mouse monoclonal anti-ASCL1 antibody (Sigma Aldrich, St. Louis, MO). Nuclei were counterstained with hematoxylin (Sigma Aldrich, St. Louis, MO).

*Flow sorting and RNA isolation*

Aberrant CD5^+^/CD19^+^ B-cells from the patient (10^6^ cells per sample) as well as peripheral blood CD19^+^ B-lymphocytes from seven healthy donors (5*10^5^ -10^6^ cells per sample) were flow sorted in 5 ml Panserin 411S medium (PAN Biotech, Aidenbach, Germany). All antibodies used were purchased from BD Biosciences (Franklin Lakes, NJ).

Flow sorting of the patient’s aberrant B-cells was performed eleven years after initial CLL diagnosis, during therapy with the Burton’s tyrosine kinase inhibitor ibrutinib.

Sorted lymphocytes were processed immediately. After centrifugation at 300 g for 8 min, cell pellets were lysed in 1 ml peqGOLD TriFast™ (Peqlab, Erlangen, Germany). After phase separation with chlorophorm, the aqueous phase was used for RNA extraction with the Direct-zol™ RNA MiniPrep Kit (Zymo Research, Irvine, CA) according to the user manual with following modifications: DNAse I Digestion was performed using the RNAse-free DNAse Set (QIAGEN, Venlo, The Netherlands) with 15 µl of DNAse I and 105 µl of the RDD buffer for 30 min at room temperature. Two extra wash steps with 400 and 700 µl Wash Buffer were performed after DNAse I digestion. RNA was eluted in 35 µl RNAse free water.

Quality control of the isolated RNA was performed before use for microarray experiments by photometric assessment of the A260/A230 and A260/A280 ratios and by chip electrophoresis with the MCE-202 MultiNA microchip electrophoresis system (Shimadzu, Kyoto, Japan). Quantitative PCR with primers for the amplification of Alu repeats was used to assess the presence of genomic DNA in the RNA samples.

*Gene expression microarray*

Array probes were prepared from total RNA samples using the OvationP^®^P Pico WTA System V2 (NuGen Technologies, San Carlos, CA). Fragmentation and labeling were performed using the EncoreP^®^P Biotin Module (Nugen Technologies, San Carlos, CA). Labeled probes were hybridized on a GeneChipP^®^P PrimeView™ Human Gene Expression Array (Affymetrix, Santa Clara, CA) according to the instructions of the manufacturer. The AffymetrixP^®^P Expression Console™ Software (v.1.4.1) (Affymetrix, Santa Clara, CA) was used for array quality control and generation of summarized expression values from the array intensity files. Gene level differential expression analysis was performed with the Transcriptome Analysis Console (TAC) 3.0 Software (Affymetrix, Santa Clara, CA) using the Tukey’s Bi-weight average algorithm. The Benjamini-Hochberg false discovery rate (FDR) procedure was used to adjust for multiple hypothesis testing.

*References*

1. Karran EL, Sonoki T, Dyer MJ. Cloning of immunoglobulin chromosomal translocations by long-distance inverse polymerase chain reaction. Methods Mol Med. 2005;115217-30.

2. Willis TG, Jadayel DM, Coignet LJ, Abdul-Rauf M, Treleaven JG, Catovsky D, et al. Rapid molecular cloning of rearrangements of the IGHJ locus using long-distance inverse polymerase chain reaction. Blood. 1997;90(6):2456-64.

3. Sonoki T, Willis TG, Oscier DG, Karran EL, Siebert R, Dyer MJ. Rapid amplification of immunoglobulin heavy chain switch (IGHS) translocation breakpoints using long-distance inverse PCR. Leukemia. 2004;18(12):2026-31.

4. Livak KJ, Schmittgen TD. Analysis of relative gene expression data using real-time quantitative PCR and the 2(-Delta Delta C(T)) Method. Methods. 2001;25(4):402-8.
