# Supplementary material for: Overexpression of the proneural transcription factor ASCL1 in chronic lymphocytic leukemia with a t(12;14)(q23.2;q32.3)
Source: Mol Cytogenet. 2018 Jan 11;11:3. doi: 10.1186/s13039-018-0355-7 (PMC5765657; doi:10.1186/s13039-018-0355-7)
Supplement: Supplementary file 2 — Primer Sequences. (DOCX 120 kb) [file 13039_2018_355_MOESM2_ESM.docx]

**Additional File 2: Table S1**

Primer sequences

| Primer name | Application | 5’-> 3’ Sequence | Reference |
| --- | --- | --- | --- |
| J6E | IGHJ LDI-PCR | CCC ACA GGC AGT AGC AGA AAA CAA | [1] |
| JBE | IGHJ LDI-PCR | GAA GCA GGT CAC CGC GAG AGT | [1] |
| JHE | IGHJ LDI-PCR | TGG GAT GCG TGG CTT CTG CT | [1] |
| JXE | IGH Sµ LDI-PCR | CAC TGG CAT CGC CCT TTG TCT AA | [1] |
| J6I | IGHJ Nested PCR | TCT GGG CTC GAG TCG ACG CAG AAA ACA AAG GCC CTA GAG GG | [1] |
| JBI | IGHJ Nested PCR | CTT CTG GTT GTG AAG AGG TGG TTT TG | [1] |
| JHI | IGHJ Nested PCR | GCC CTT GTT AAT GGA CTT GGA GGA | [1] |
| JXI | IGH Sµ Nested PCR | CCC ATG CCT TCC AAA GCG ATT | [1] |
| SAE | IGH Sµ LDI-PCR | ACA TAA ATG AGT CTC CTG CTC TTC ATC AAG | [2] |
| SAI | IGH Sµ Nested PCR | GCA ATT AAG ACC AGT TCC CCT TCT AGT G | [2] |
| γF1 | IGH Sγ LDI-PCR | TCC CTG AGG TGG CAC CGA TG | [2] |
| γF4 | IGH Sγ LDI-PCR | CAC GCA GAA GAG CCT CTC CCT GT | [2] |
| γR1 | IGH Sγ LDI-PCR | GAC CAG TGG ACA CTG TTC TCA GAT GG | [2] |
| γF2 | IGH Sγ Nested PCR | CCA GAG CTG AGG CCA AGC TAG AG | [2] |
| γF5 | IGH Sγ Nested PCR | CCC AGC ATG GAA ATA AAG CAC CC | [2] |
| γR2 | IGH Sγ Nested PCR | CCT CCA AGG CCC TTT TCT TCT GTG | [2] |
| αF1 | IGH Sα LDI-PCR | GCA CAC TGA GTG TCA GAC CCA GTC TC | [2] |
| αR1 | IGH Sα LDI-PCR | AGC ACA GAG AGG CCT GGT GAC AG | [2] |
| αF2 | IGH Sα Nested PCR | CGG GAC CCA GTC ACT GAA TAC GT | [2] |
| αR2 | IGH Sα Nested PCR | TGG TTT CTG AAC ATG CTC CTT AGA TAG G | [2] |
| IGH der12 Rv | der(12) BP seq | TGG CAA TGA GAT GGC TTT AGC TGA GA | Present study |
| IGH der14 Fw | der(14) BP seq | TCA GCC CCA GCC CAG GTT AG | Present study |
| IGH der14 Rv | der(14) BP seq | CCT CCC CTT GGA AAC TCC GT | Present study |

**References**

1. Willis TG, Jadayel DM, Coignet LJ, Abdul-Rauf M, Treleaven JG, Catovsky D, et al. Rapid molecular cloning of rearrangements of the IGHJ locus using long-distance inverse polymerase chain reaction. Blood. 1997;90(6):2456-64.

2. Sonoki T, Willis TG, Oscier DG, Karran EL, Siebert R, Dyer MJ. Rapid amplification of immunoglobulin heavy chain switch (IGHS) translocation breakpoints using long-distance inverse PCR. Leukemia. 2004;18(12):2026-31.
