# Supplementary material for: Overexpression of the proneural transcription factor ASCL1 in chronic lymphocytic leukemia with a t(12;14)(q23.2;q32.3)
Source: Mol Cytogenet. 2018 Jan 11;11:3. doi: 10.1186/s13039-018-0355-7 (PMC5765657; doi:10.1186/s13039-018-0355-7)
Supplement: Supplementary file 3 — Hierarchical Clustering. (DOCX 115 kb) [file 13039_2018_355_MOESM3_ESM.docx]

Additional File 3: Figure S1


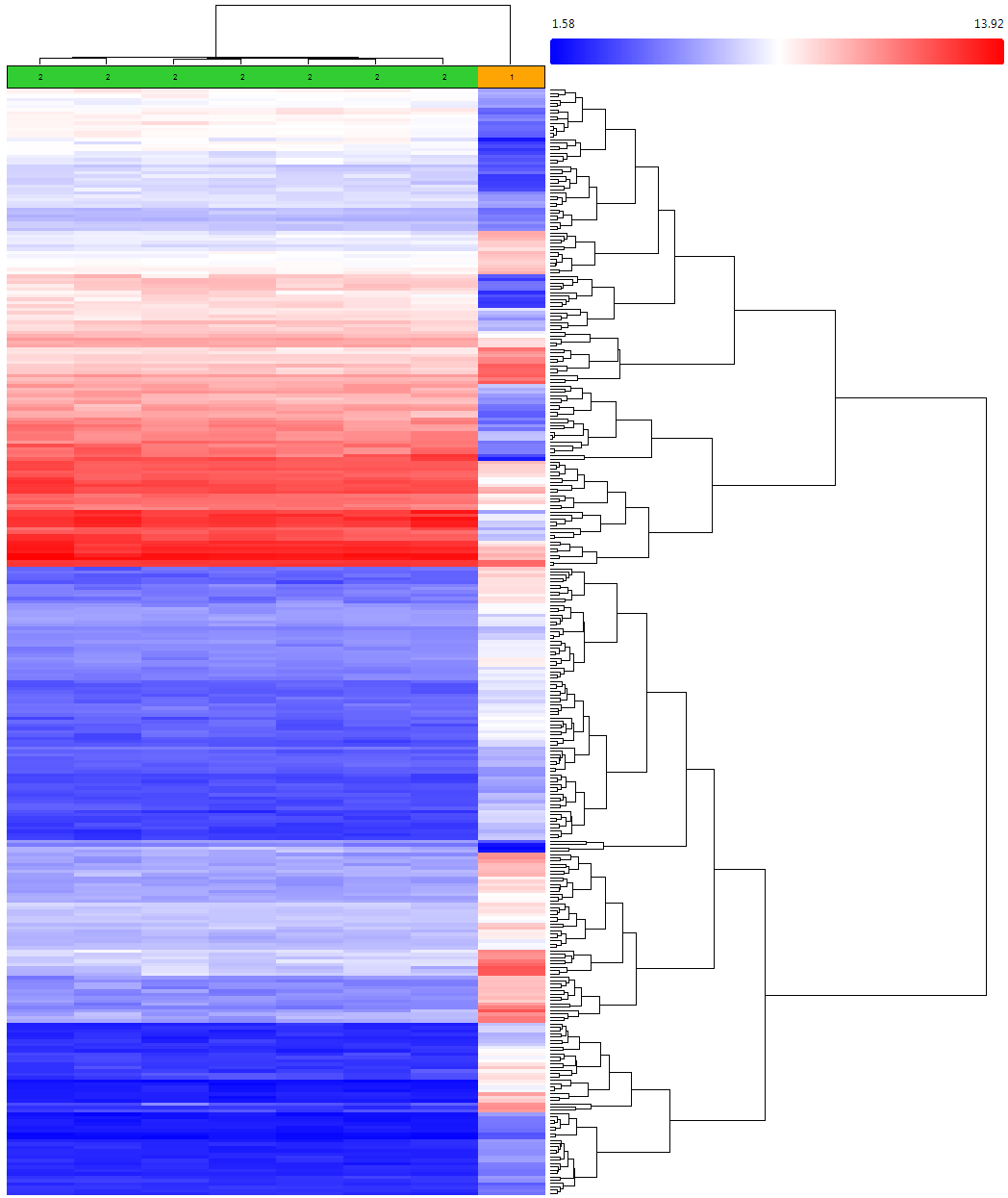


**Additional Figure S1** Hierarchical clustering (TAC 3.0 Software, Affymetrix) of the patient (right, highlighted in orange) and the seven control (left, highlighted in green) samples. Transcripts with FC ≥ 2 and FDR p ≤ 0.01 are depicted.
