# Supplementary material for: Overexpression of the proneural transcription factor ASCL1 in chronic lymphocytic leukemia with a t(12;14)(q23.2;q32.3)
Source: Mol Cytogenet. 2018 Jan 11;11:3. doi: 10.1186/s13039-018-0355-7 (PMC5765657; doi:10.1186/s13039-018-0355-7)
Supplement: Supplementary file 4 — List of deregulated genes (Fold Change ≥2, FDR p ≤ 0.01). (DOCX 474 kb) [file 13039_2018_355_MOESM4_ESM.docx]

**Additional File 4: Table S2**

List of the deregulated genes in the patient (Fold Change ≥ 2, FDR p ≤ 0.01). References are provided for genes whose deregulation is concordant to other studies comparing normal to CLL B-cells. Potential transcriptional ASCL1 targets are indicated by the respective citations.

| Transcript Cluster ID | Fold Change | FDR p-value | Gene Symbol | Reference ASCL1 | Reference CLL |
| --- | --- | --- | --- | --- | --- |
| 11757864_s_at | 209.42 | 0.000137 | *INSM1* | [1, 2] |  |
| 11726674_at | 202.32 | 0.000029 | *ASCL1* |  |  |
| 11730520_a_at | 118.18 | 0.000114 | *ABCA9* |  | [3-5] |
| 11735862_at | 110.69 | 0.008387 | *DLEU7* | [1] |  |
| 11757480_x_at | 97.85 | 0.002866 | *IFI27* |  |  |
| 11739904_a_at | 97.65 | 0.001197 | *ABCA6* |  | [3-6] |
| 11753066_a_at | 84.87 | 0.000166 | *PAH* |  |  |
| 11740888_a_at | 82.52 | 0.000408 | *MRO* |  |  |
| 11716109_a_at | 54.68 | 0.007529 | *PIGR* |  | [3-5] |
| 11733243_a_at | 42.47 | 0.000495 | *GABRB2* |  | [4] |
| 11740865_at | 41.65 | 0.003324 | *CLEC9A* |  |  |
| 11733948_at | 38.71 | 0.0008 | *PAX9* |  |  |
| 11740230_a_at | 36.72 | 0.004427 | *PHEX* |  | [4, 5] |
| 11716826_a_at | 34.95 | 0.001694 | *FMOD* |  | [3-6] |
| 11727675_at | 34.11 | 0.006301 | *MOCOS* |  |  |
| 11730924_at | 31.31 | 0.004063 | *AICDA* |  |  |
| 11738285_at | 31.25 | 0.005326 | *CLNK* |  | [4, 5] |
| 11730677_a_at | 27.45 | 0.000748 | *RNF150* |  |  |
| 11743306_at | 25.51 | 0.000537 | *FHDC1* |  | [3-5] |
| 11723181_a_at | 24.93 | 0.002038 | *MAPK4* |  |  |
| 11756285_s_at | 23.16 | 0.007352 | *IGF2BP3* |  | [5] |
| 11724641_a_at | 21.43 | 0.001351 | *ARHGAP44* |  | [3-6] |
| 11734294_at | 20.52 | 0.000458 | *KCNJ11* |  | [4, 5] |
| 11743720_s_at | 20.37 | 0.000748 | *KSR2* | [7] | [3-5] |
| 11754924_s_at | 20.35 | 0.007292 | *ADTRP* |  | [3, 5] |
| 11732758_a_at | 20.04 | 0.008325 | *POF1B* |  |  |
| 11756789_a_at | 19.58 | 0.008732 | *ATRNL1* |  |  |
| 11725040_at | 18.37 | 0.000748 | *FGF2* |  |  |
| 11731172_s_at | 18.02 | 0.000881 | *GLDN* |  |  |
| 11720279_a_at | 16.98 | 0.006932 | *FAM174B* |  | [5] |
| 11727280_a_at | 16.57 | 0.008957 | *KCNH2* | [1, 2] | [3, 4] |
| 11742206_a_at | 16.02 | 0.006301 | *CLLU1* |  | [4, 5] |
| 11715353_s_at | 15.23 | 0.002046 | *COL1A2* |  |  |
| 11733756_a_at | 14.87 | 0.004427 | *VWC2L* |  |  |
| 11751533_a_at | 14.09 | 0.002923 | *FRK* |  |  |
| 11719026_a_at | 13.81 | 0.002665 | *PSD3* |  |  |
| 11720372_at | 13.5 | 0.007529 | *TESC* |  | [3-5] |
| 11737644_a_at | 12.83 | 0.002621 | *RNF183* | [7] |  |
| 11715109_at | 12.79 | 0.006586 | *SAMD7* |  |  |
| 11725382_a_at | 12.62 | 0.002736 | *RAPGEF3* | [1, 8] | [3-5] |
| 11747224_s_at | 12.07 | 0.000495 | *SFTPB* |  | [4, 5] |
| 11722855_at | 11.83 | 0.001746 | *PDGFD* |  | [3-5] |
| 11761892_at | 10.74 | 0.002153 | *LINC00334* |  |  |
| 11761824_at | 10.65 | 0.001541 | *NSAP11* |  |  |
| 11729887_at | 10.59 | 0.00115 | *PTPLA* |  | [3, 4] |
| 11737658_at | 10.03 | 0.002655 | *TMEM244* |  |  |
| 11721399_a_at | 9.91 | 0.000845 | *SLIT2* |  |  |
| 11740141_a_at | 9.63 | 0.002762 | *EPB41L1* |  |  |
| 11743224_a_at | 9.63 | 0.001307 | *IQSEC3* |  |  |
| 11735640_a_at | 9.39 | 0.00364 | *DNAH14* |  |  |
| 11729590_s_at | 9.17 | 0.005021 | *KLK2* |  | [4, 5] |
| 11755908_s_at | 8.37 | 0.006301 | *IGSF3* | [8] | [3, 4] |
| 11725590_a_at | 8.06 | 0.006774 | *NUDT6* |  | [3] |
| 11748570_a_at | 7.92 | 0.007695 | *FAXDC2* |  |  |
| 11719092_at | 7.67 | 0.007445 | *DUSP26* |  |  |
| 11723363_at | 7.64 | 0.003296 | *L1TD1* |  | [5] |
| 11759206_at | 7.53 | 0.002736 | *PON1* |  |  |
| 11760582_a_at | 7.46 | 0.000748 | *NOSIP* |  | [3-5] |
| 11726933_at | 7.34 | 0.004684 | *SOX12* |  | [4] |
| 11761325_at | 6.82 | 0.005077 | *ARHGAP21* |  | [5] |
| 11729001_a_at | 6.48 | 0.003699 | *TBX15* |  |  |
| 11759930_at | 6.26 | 0.007267 | *PHTF1* |  | [3-5] |
| 11750774_x_at | 6.24 | 0.001739 | *FBXO27* |  | [5] |
| 11732876_at | 6.23 | 0.004728 | *TRPM8* |  |  |
| 11735592_a_at | 5.81 | 0.001941 | *MYO3B* |  |  |
| 11727347_at | 5.55 | 0.003017 | *ITPKA* |  | [3, 5] |
| 11718544_s_at | 5.34 | 0.00999 | *ZBTB44* |  |  |
| 11725357_a_at | 5.19 | 0.003376 | *PRTFDC1* |  |  |
| 11740889_a_at | 5.16 | 0.007229 | *ATP2A1* |  |  |
| 11725685_a_at | 5.13 | 0.006616 | *PTPRO* |  | [4] |
| 11731574_at | 4.96 | 0.006301 | *FRMD4B* |  | [5] |
| 11727427_a_at | 4.94 | 0.000698 | *ACSM3* |  | [5] |
| 11761540_x_at | 4.84 | 0.008732 | *C20orf96* |  | [4] |
| 11728636_s_at | 4.62 | 0.006462 | *DPPA3; DPPA3P2* |  |  |
| 11740603_a_at | 4.58 | 0.002016 | *APH1B* |  | [4, 5] |
| 11722074_s_at | 4.54 | 0.005445 | *HEYL* |  |  |
| 11737395_at | 4.5 | 0.004973 | *SOWAHD* |  | [5] |
| 11730396_at | 4.35 | 0.005021 | *DDI1* |  |  |
| 11723928_at | 4.31 | 0.001694 | *PNMA2* |  | [5] |
| 11757273_s_at | 4.2 | 0.005199 | *MBP* | [1] | [4, 5] |
| 11748972_a_at | 4.15 | 0.004976 | *CDC25B* | [2, 8] | [4] |
| 11717359_at | 4.13 | 0.003324 | *RTF1* |  |  |
| 11728105_x_at | 4.04 | 0.001197 | *TMPRSS3* |  |  |
| 11734055_a_at | 3.89 | 0.000489 | *C20orf26* |  |  |
| 11754570_a_at | 3.88 | 0.006522 | *GNB3* |  |  |
| 11759282_x_at | 3.78 | 0.003699 | *SERINC5* |  | [3-5] |
| 11737177_at | 3.78 | 0.004271 | *SLC25A21* |  |  |
| 11719164_a_at | 3.69 | 0.006301 | *CLCN5* |  |  |
| 11744558_a_at | 3.59 | 0.00386 | *VASH1* | [8] | [4, 5] |
| 11744038_a_at | 3.5 | 0.008245 | *NKAIN4* |  |  |
| 11740566_at | 3.45 | 0.001307 | *ATXN3L* |  |  |
| 11730060_a_at | 3.44 | 0.00472 | *LAG3* |  | [3] |
| 11728236_at | 3.43 | 0.008872 | *CD5* |  | [4, 5] |
| 11732272_at | 3.37 | 0.008774 | *ANGPTL3* |  |  |
| 11718570_at | 3.33 | 0.003773 | *NT5DC3* |  |  |
| 11723574_a_at | 3.13 | 0.00629 | *MORC4* |  |  |
| 11736843_a_at | 3.12 | 0.005213 | *KLRAP1* |  |  |
| 11737263_at | 3.1 | 0.004849 | *HRK* |  |  |
| 11757949_s_at | 3.06 | 0.001391 | *NINL* | [8] | [4, 5] |
| 11724325_a_at | 2.84 | 0.009454 | *RABGAP1L* |  |  |
| 11734389_a_at | 2.66 | 0.003163 | *GLYATL2* |  |  |
| 11735377_at | 2.54 | 0.009344 | *MSTN* |  |  |
| 11740902_at | 2.47 | 0.005326 | *PKD1L1* |  |  |
| 11743779_at | 2.43 | 0.009649 | *HERC2* |  |  |
| 11760325_x_at | 2.32 | 0.002819 | *MSI2* |  | [4, 5] |
| 11755195_a_at | 2.24 | 0.009098 | *NXPE1* |  |  |
| 11730335_a_at | 2.15 | 0.004728 | *PARP3* |  | [4] |
| 11734719_a_at | 2.13 | 0.005442 | *DNAJC14* |  |  |
| 11759758_at | 2.08 | 0.00999 | *LOC339803* |  |  |
| 11731473_at | 2.06 | 0.007055 | *TMEM70* |  | [5] |
| 11750908_a_at | -2.33 | 0.004973 | *ESR2* |  | [3] |
| 11720117_a_at | -2.35 | 0.006529 | *SIPA1* |  | [3, 4, 6] |
| 11734149_x_at | -2.37 | 0.006601 | *PRRT3* |  |  |
| 11760774_x_at | -2.49 | 0.005484 | *GNL1* |  |  |
| 11718164_at | -2.52 | 0.009344 | *PPP1R15B* |  | [4] |
| 11739796_a_at | -2.53 | 0.002447 | *RHBDF2* |  | [3-5] |
| 11727416_a_at | -3.07 | 0.009949 | *CRYL1* |  |  |
| 11743053_a_at | -3.27 | 0.005613 | *CHORDC1* |  |  |
| 11747152_a_at | -3.51 | 0.003528 | *PHLDB2* |  |  |
| 11722721_a_at | -3.57 | 0.009454 | *CENPV* |  | [5] |
| 11732077_at | -3.75 | 0.005445 | *IGSF22* |  |  |
| 11755843_a_at | -3.83 | 0.006984 | *PCNXL2* | [7] |  |
| 11721965_a_at | -4.05 | 0.008131 | *NOTCH4* |  |  |
| 11715413_s_at | -4.39 | 0.004234 | *TSC22D1* |  | [3] |
| 11734583_a_at | -4.47 | 0.006774 | *ZDHHC14* | [8] | [3-5] |
| 11753899_a_at | -5.06 | 0.006377 | *DLEU1* |  |  |
| 11732996_at | -5.1 | 0.007435 | *HSP90AA1* |  | [4] |
| 11753179_s_at | -5.38 | 0.004207 | *FAM134B* |  | [3, 5] |
| 11758106_at | -5.42 | 0.00556 | *NRM* |  | [3] |
| 11721621_a_at | -5.87 | 0.004728 | *KATNAL1* |  | [3] |
| 11734215_x_at | -5.93 | 0.007847 | *MPP7* |  |  |
| 11716710_a_at | -5.98 | 0.007898 | *ADM* |  |  |
| 11757596_s_at | -6.15 | 0.009642 | *TRIM13* |  | [4] |
| 11759525_at | -7.07 | 0.000458 | *GADD45B* |  | [3] |
| 11756780_a_at | -7.45 | 0.006015 | *MS4A7* |  |  |
| 11755166_a_at | -7.83 | 0.008895 | *ESAM* |  | [3, 5] |
| 11740912_a_at | -8.64 | 0.005102 | *BANK1* |  | [3-5] |
| 11717337_a_at | -8.95 | 0.004432 | *ITGB2* |  | [5] |
| 11732581_a_at | -9.32 | 0.002321 | *SLC24A5* |  |  |
| 11730469_s_at | -9.57 | 0.007944 | *LMO2* | [8] | [5] |
| 11748244_a_at | -12.59 | 0.002229 | *ZNF300* |  | [3] |
| 11755520_s_at | -12.7 | 0.001739 | *CERS6* |  | [3] |
| 11729976_a_at | -12.96 | 0.004208 | *PTPRN2* | [7] | [3, 5] |
| 11721577_at | -12.99 | 0.005021 | *TNF* |  | [4] |
| 11723727_a_at | -14.55 | 0.00629 | *CXorf57* |  | [3-5] |
| 11717132_s_at | -16.23 | 0.006387 | *AHNAK2* |  | [5] |
| 11727732_a_at | -16.97 | 0.005445 | *LCN10* |  | [5] |
| 11744413_x_at | -17.98 | 0.001207 | *HSPA1A;HSPA1B* |  |  |
| 11720159_a_at | -18.34 | 0.002866 | *DENND3* |  | [3-5] |
| 11715446_a_at | -19.42 | 0.004234 | *DNAJB1* |  |  |
| 11721702_a_at | -20.4 | 0.004727 | *CMTM7* |  | [3-5] |
| 11716151_a_at | -20.95 | 0.005445 | *PMEPA1* |  | [3, 4] |
| 11752200_x_at | -21.43 | 0.000489 | *APP* |  |  |
| 11734955_a_at | -21.5 | 0.005213 | *SCML1* |  | [3-5] |
| 11724378_s_at | -21.52 | 0.001694 | *PAG1* |  | [3-5] |
| 11733698_s_at | -22.24 | 0.001593 | *SGK1* | [1] | [4] |
| 11715757_a_at | -24.38 | 0.000902 | *RGS2* | [1] | [3, 4] |
| 11754518_a_at | -25.66 | 0.00999 | *NEIL1* | [1] | [3, 4] |
| 11727996_a_at | -28.31 | 0.005732 | *MYEF2* |  |  |
| 11743001_a_at | -30.21 | 0.007267 | *ADD2* |  | [3-5] |
| 11717345_a_at | -36.54 | 0.001541 | *FOSB* |  | [4, 5] |
| 11756992_a_at | -39.55 | 0.005021 | *MARC2* |  | [3] |
| 11746463_a_at | -40 | 0.008895 | *IL6* |  | [3-5] |
| 11720875_at | -45.12 | 0.00773 | *CORO2B* | [1, 8] | [3] |
| 11715306_s_at | -46.5 | 0.00115 | *AREG* |  | [3] |
| 11749291_a_at | -47.09 | 0.008732 | *FOS* | [1, 7] | [5] |
| 11720153_s_at | -53.16 | 0.001682 | *NRIP1* |  | [3-6] |
| 11726800_at | -61.42 | 0.000976 | *EBF1* |  | [3-6] |
| 11754145_x_at | -61.59 | 0.0017 | *IGKV1-27 IGKV1D-27* |  |  |
| 11719560_at | -63.06 | 0.002665 | *SOX4* | [1] | [3-5] |
| 11722359_x_at | -66.55 | 0.001207 | *EPB41L2* |  | [3-5] |
| 11725632_at | -72.9 | 0.004063 | *NR4A2* |  | [3, 4] |
| 11716551_s_at | -85.6 | 0.004498 | *HES1* |  | [3, 4] |
| 11748396_x_at | -92.99 | 0.003163 | *MYADM* |  | [3-5] |
| 11763678_x_at | -97.97 | 0.002621 | *IGHM* |  | [6] |
| 11742765_at | -98.36 | 0.00097 | *RGS1* |  | [3] |

**REFERENCES**

1. Castro DS, Martynoga B, Parras C, Ramesh V, Pacary E, Johnston C, et al. A novel function of the proneural factor Ascl1 in progenitor proliferation identified by genome-wide characterization of its targets. Genes Dev. 2011;25(9):930-45.

2. Borromeo MD, Meredith DM, Castro DS, Chang JC, Tung KC, Guillemot F, et al. A transcription factor network specifying inhibitory versus excitatory neurons in the dorsal spinal cord. Development. 2014;141(14):2803-12.

3. Ruiz-Lafuente N, Alcaraz-Garcia MJ, Sebastian-Ruiz S, Gomez-Espuch J, Funes C, Moraleda JM, et al. The gene expression response of chronic lymphocytic leukemia cells to IL-4 is specific, depends on ZAP-70 status and is differentially affected by an NFkappaB inhibitor. PLoS One. 2014;9(10):e109533.

4. Ferreira PG, Jares P, Rico D, Gomez-Lopez G, Martinez-Trillos A, Villamor N, et al. Transcriptome characterization by RNA sequencing identifies a major molecular and clinical subdivision in chronic lymphocytic leukemia. Genome Res. 2014;24(2):212-26.

5. Liao W, Jordaan G, Nham P, Phan RT, Pelegrini M, Sharma S. Gene expression and splicing alterations analyzed by high throughput RNA sequencing of chronic lymphocytic leukemia specimens. BMC Cancer. 2015;15714.

6. Jelinek DF, Tschumper RC, Stolovitzky GA, Iturria SJ, Tu Y, Lepre J, et al. Identification of a global gene expression signature of B-chronic lymphocytic leukemia. Mol Cancer Res. 2003;1(5):346-61.

7. Augustyn A, Borromeo M, Wang T, Fujimoto J, Shao C, Dospoy PD, et al. ASCL1 is a lineage oncogene providing therapeutic targets for high-grade neuroendocrine lung cancers. Proc Natl Acad Sci U S A. 2014;111(41):14788-93.

8. Raposo AA, Vasconcelos FF, Drechsel D, Marie C, Johnston C, Dolle D, et al. Ascl1 Coordinately Regulates Gene Expression and the Chromatin Landscape during Neurogenesis. Cell Rep. 2015.
